# Corrective Osteotomy for Coronal Plane Malunion of the Medial Femoral Condyle

**DOI:** 10.7759/cureus.3222

**Published:** 2018-08-28

**Authors:** Fırat Ozan, Kürşat Tuğrul Okur, Ömer Can Ünlü, Muhammed Melez, Kamil Yamak, Cemil Kayalı, Taşkın Altay

**Affiliations:** 1 Orthopedics and Traumatology, Kayseri Training and Research Hospital, Kayseri, TUR; 2 Orthopedics and Traumatology, Kayseri Education and Research Hospital, Kayseri, TUR; 3 Orthopedics and Traumatology, İzmir Bozyaka Training and Research Hospital, Izmir, TUR; 4 Orthopedics and Traumatology, İzmir Bozyaka Training and Research Hospital, İzmir, TUR

**Keywords:** coronal fracture of femur condyles, malunion, hoffa fracture, corrective osteotomy

## Abstract

Medial femoral condyle malunion in the coronal plane is a very rare injury. In this presented case, we performed intra-articular corrective osteotomy for a malunited medial femoral condyle in the coronal plane of a 22-year-old man and obtained good functional and radiographic results. Corrective osteotomy for a malunited medial Hoffa fracture is technically very challenging, but intra-articular corrective osteotomy for these malunited fractures offers a good outcome and should be considered as a salvage treatment.

## Introduction

Coronal plane fractures of the femoral condyles are rare intra-articular injuries [1]. Isolated fractures of the femoral condyle represent only 0.65% of all femoral fractures [2]. The first known description of a coronal fracture of the lateral femoral condyle was published by Busch in 1869. Hoffa used Busch's drawing in the first edition of his book in 1888. But almost all publications cite the fourth edition of Hoffa's book in 1904, presenting this date as the year when a fracture of the posterior femoral condyle was initially described [1].

The lateral condyle is injured three times more frequently than the medial one. The prepotency of the lateral condyle can be attributed to physiological valgus at the knee [3-4]. Generally, Hoffa fractures are seen as a result of a high-velocity, high-energy trauma [5]. The mechanism of injury that produces a coronal plane fracture of the femoral condyle is unknown, but a shearing force on the posterior femoral condyle is assumed [3,6]. However, the mechanism of injury for a coronal plane fracture of the medial femoral condyle has reported that a direct impact on the medial side with the knee at 90° of flexion can also cause trauma [3,5-7].

Treating these fractures is important to achieve good anatomical reduction of the articular surface. Following surgery, an early rehabilitation program is recommended for better functional outcomes. Non-operative management of these fractures generally results in poor outcomes, including malunion, non-union, and avascular necrosis [5,8-9].

An isolated medial femoral condyle fracture in the coronal plane is an extremely rare injury, but medial femoral condyle malunion in the coronal plane is a more rare injury [2-5]. We identified only one case report in the literature.

Corrective osteotomy for medial femoral condyle malunion in the coronal plane is an acceptable salvage option, as it can reduce pain, improve the range of motion of the knee, and prevent joint damage and post-traumatic arthritis in the future [4].

This case report highlights the approach to a case of applied intra-articular corrective osteotomy for medial femoral condyle malunion in the coronal plane.

## Case presentation

**Figure 1:**
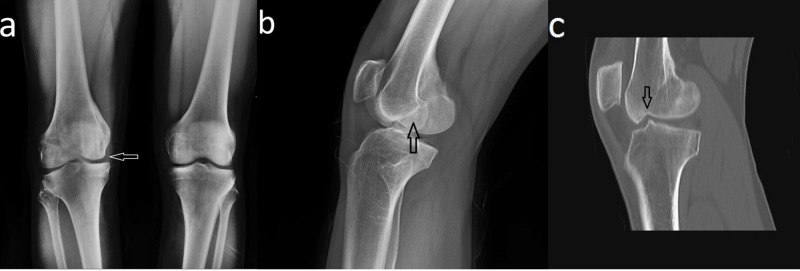
Pre-operative radiographic images of the patient (a,b) Anteroposterior and lateral radiographic images of the patient show a coronal plane fracture (type III) of the medial femoral condyle of the right knee (arrow). (c) Computed tomography image shows the incongruity of the articular surface (arrow).

A 22-year-old male patient presented to our polyclinic with pain, deformity, and limited joint mobility in the right knee. He suffered a fall about three years ago and did not receive any kind of treatment. A physical examination showed a 10° varus deformity, a 25° flexion contracture, and a limited amount of joint movement in the patient's right knee. There was no neurological damage. Radiographs and computed tomography (CT) images showed a malunited isolated medial condyle fracture in the coronal plane with an intra-articular incongruity (Figure 1).

**Figure 2:**
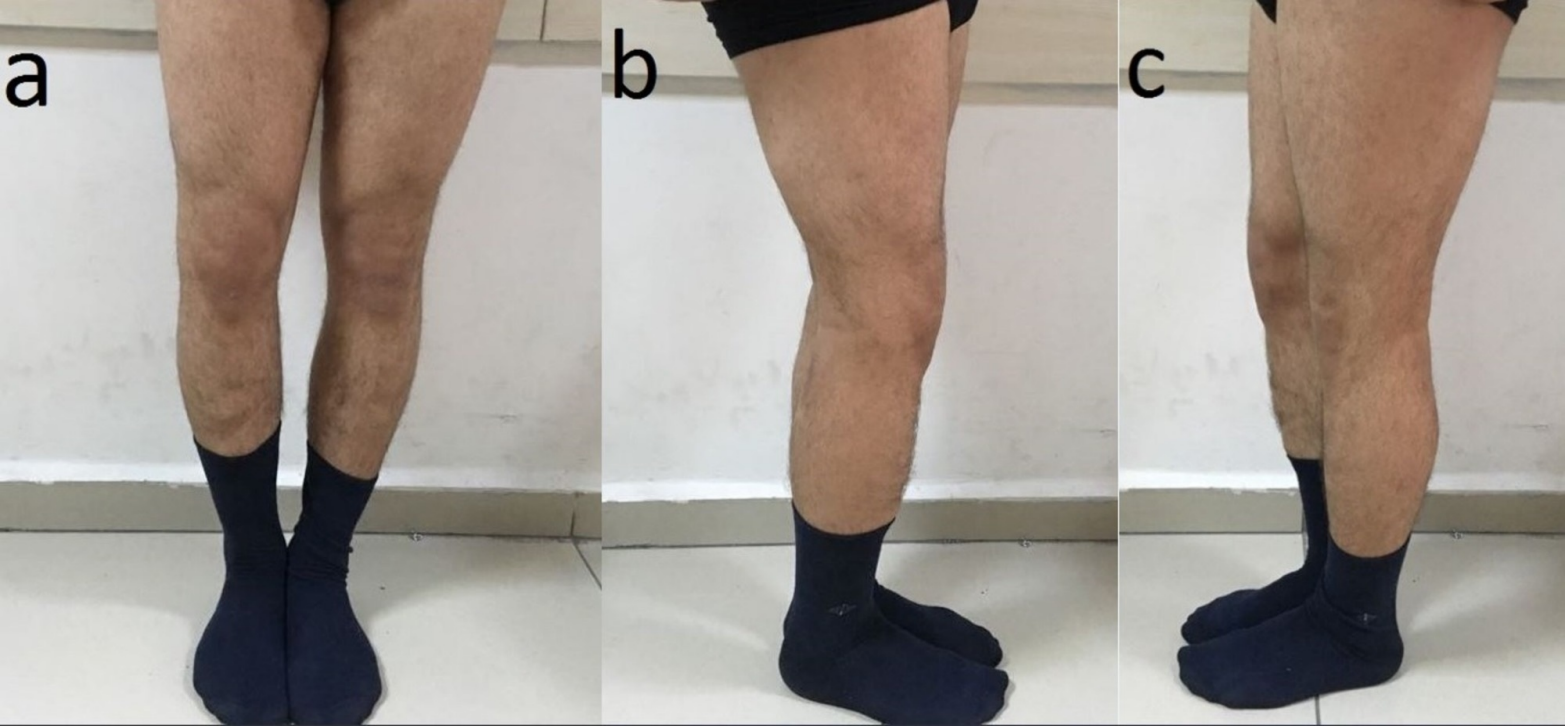
The appearance of deformity (a,b,c) The appearance of varus deformity and flexion contracture in the right knee of the patient.

Magnetic resonance imaging (MRI) revealed no ligaments injury in the knee. We planned for corrective osteotomy of the medial femoral condyle (Figure 2).

The knee was placed in the flexed position for skin incision. An anterior skin incision was made that extends 3 cm proximal to the patella to the tibial tubercle. The medial parapatellar arthrotomy was made 2 cm proximal to the patella, curving along the medial patella and parallel to the patellar ligament to the tibial tubercle, and the distal medial femoral condyle was exposed. An approximately 5-mm step was detected in the medial femoral condyle. The chondral structures, meniscus, and ligaments were in good shape. An osteotomy line was identified with fluoroscopy using two Kirschner wires. Then, corrective osteotomy was carried out carefully. Posterior soft tissue dissection was not performed to protect the blood supply of the femur medial condyle. Therefore, a difficulty was encountered in bringing the osteotomized medial condyle to an anatomical position. This problem has been overcome by hyperflexing the knee and letting the tibial plateau push the medial condyle forward.

**Figure 3:**
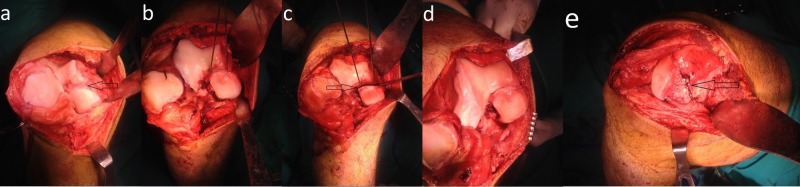
Intraoperative view of the patient (a) Intraoperative view of the defect in the medial femoral condyle after the medial parapatellar arthrotomy (arrow); (b,c) Identification of the osteotomy line with Kirschner wires and the application of the corrective osteotomy (arrow); (d,e) View of the medial femoral condyle after reduction and screws fixation (arrow).

The condylar osteotomy fragment was fixed by inserting two 4.5-mm headless compression screws from the anterior to the posterior direction of the medial femoral condyle. Then, another screw was inserted from the medial to the lateral direction. The joint movements were checked, and it was found that the varus deformities of the knee improved. Finally, the exposure was closed (Figure 3).

**Figure 4:**
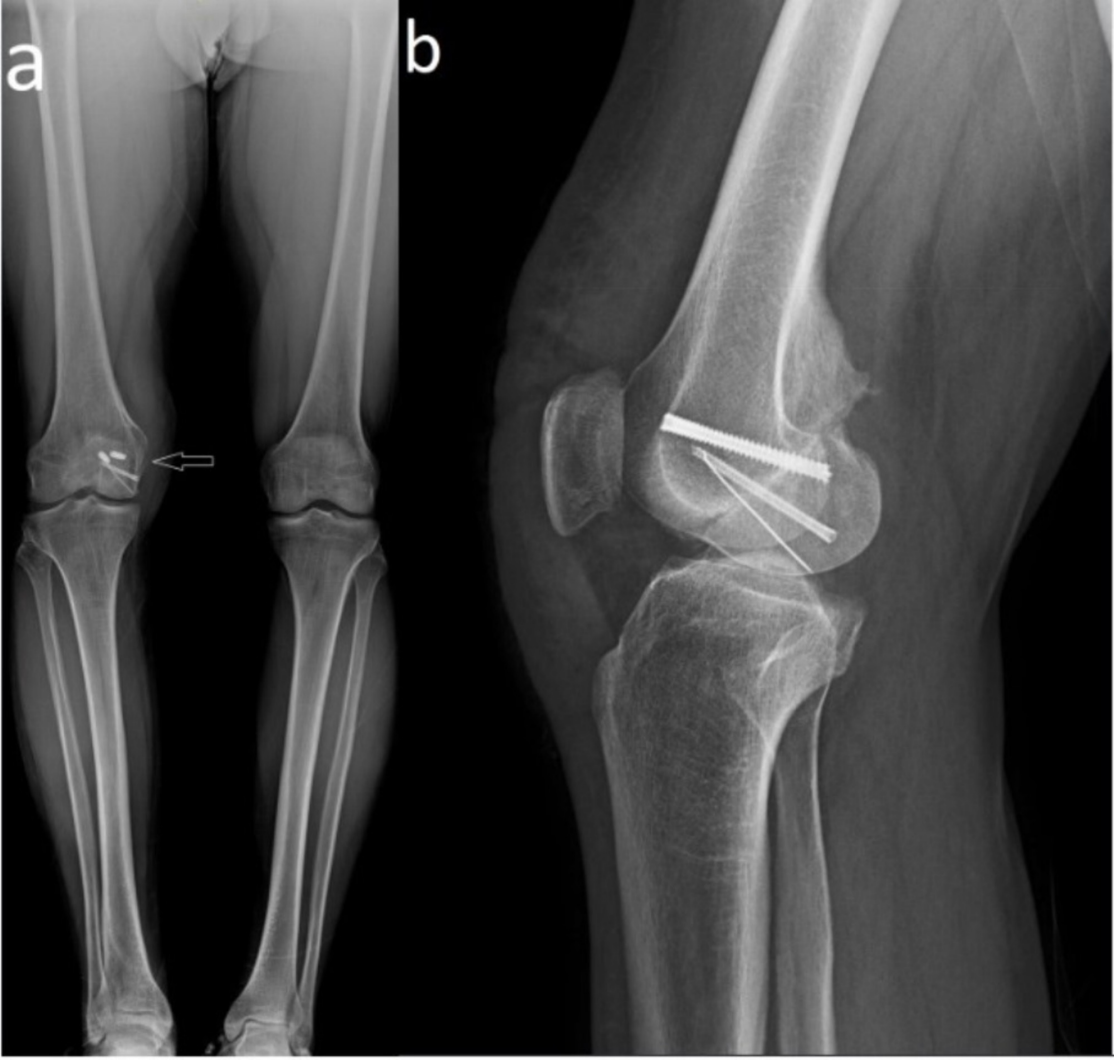
Radiographic images at the end of the follow-up (a) Anteroposterior orthoroentgenogram (arrow) and (b) lateral radiographic images at the end of the follow-up of the patient.

Postoperatively, a long-leg splint was applied to the knee joint with ﬂexion at 30°. The cast was applied for two weeks (Figure 4).

**Figure 5:**
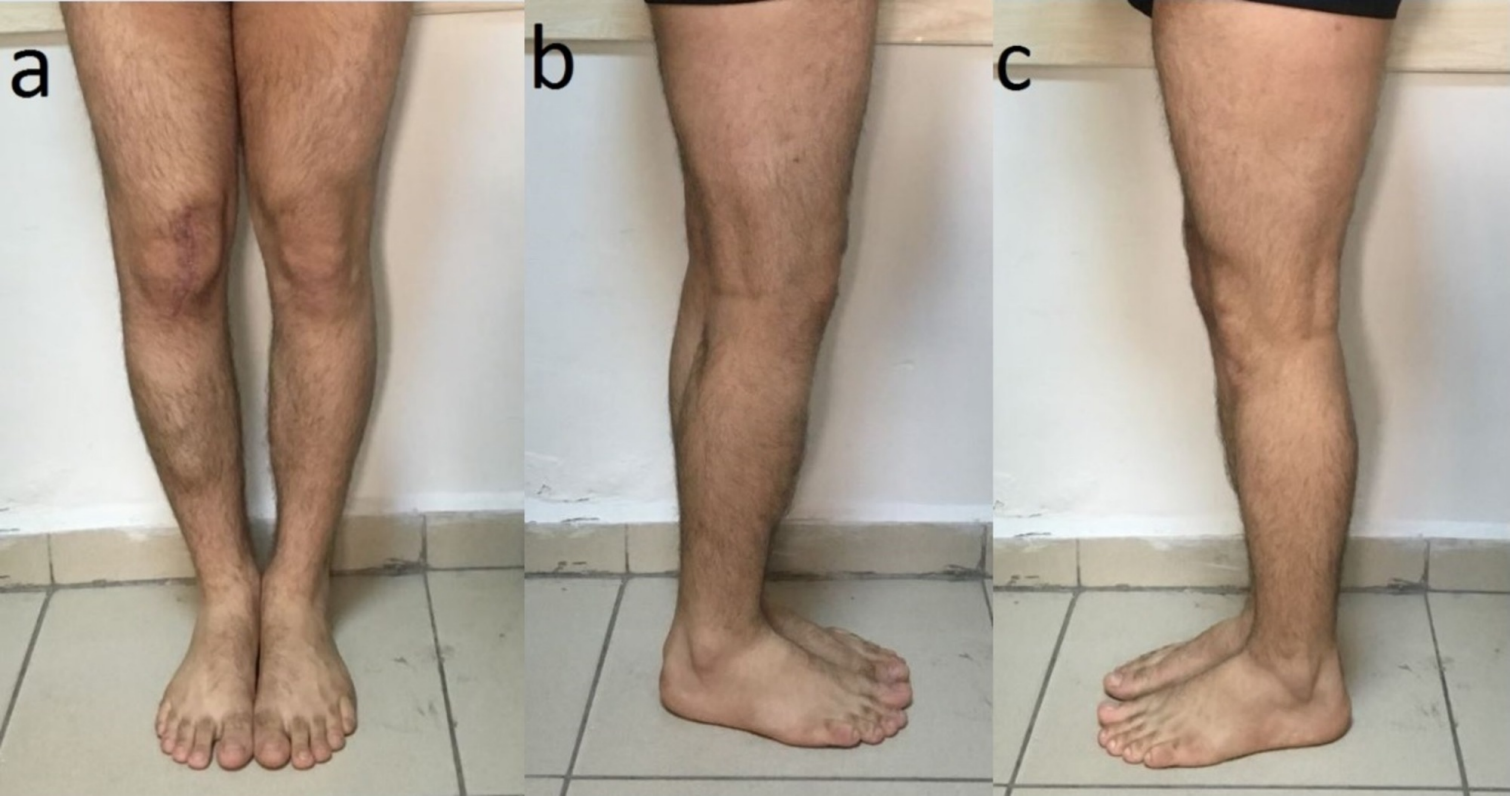
Post-operative extremity images (a,b,c) Post-operative extremity images of the patient showed that the deformity of the right knee has been corrected.

Then, physical therapy was initiated such as active and passive joint movements and other muscle-strengthening exercises. The patient was allowed to bear weight as tolerated after two months. During the nine-month follow-up, the patient returned to his normal activities. The functional outcome of the patient and knee joint alignment were improved without pain or disability (Figure 5).

This study was conducted in accordance with the ethical guidelines of the Declaration of Helsinki. The patient provided written informed consent before participation.

## Discussion

Intra-articular coronal plane fractures of the distal femur medial condyle are unstable fractures. Up to 31% of these rare injuries is overlooked on plain radiographs [3,5,7,10]. Even though an oblique radiograph may be helpful, a CT scan is generally recommended to accurately identify the coronal fractures [5,10-11]. Also, a CT scan is critical for pre-operative planning, with regard to the surgical approach and implant selection [3].

Coronal plane fractures of the medial femoral condyle, otherwise known as Hoffa fractures, are classified as a Type 33-B3 fracture by the Orthopaedic Trauma Association [12]. Another classification system, as reported by Letenneur et al., divides Hoffa fractures into three types based on the distance of the fracture line from the posterior cortex of the femoral shaft [13]. Type I is a vertical fracture involving the entire condyle parallel to the posterior cortex of the femur. Type II is a fracture of variable size horizontal to the base of the condyle. Type III is a fracture oblique to the femur and has the worst results. On the basis of this classification, our patient had a Type III fracture [13]. In a cadaveric study, Lewis et al. classified the Hoffa fractures into three types. In Type I and Type III Hoffa fractures, some soft tissue elements remain attached to the fractured condylar fragment to provide blood supply [14]. But in Type II Hoffa fractures, no soft tissue element was attached to the fractured condylar fragment [14].

One of the most important differences between the medial and lateral femoral condyles is that the intraosseous blood supply to the medial femoral condyle has limited vascularity compared with that of the lateral femoral condyle [4-5]. The arterial supply of the medial femoral condyle is primarily contributed by the superior medial genicular artery, but the lateral femoral condyle is supplied by both superior and inferior lateral genicular arteries [5]. Therefore, we did not dissect the posterior surface of the medial femoral condyle to avoid osteonecrosis.

Surgical open reduction and internal fixation were performed to achieve a good outcome for coronal plane fractures of the femoral condyle [4,6]. Although it is agreed that surgical fixation is the preferred method of treatment for these fractures, there is no consensus on the surgical approach, mode of fixation, type of implant, rehabilitation protocol, and outcome measures [4,6,9]. Failure of fixation, non-union and malunion are common complications of this unstable fracture pattern.

Usually, an anterolateral or anteromedial incision is used depending on the condyle involved [7]. It has been recommended that the approach selected should depend on the fracture configuration [15]. In the presented case, we performed the medial parapatellar arthrotomy with an anterior skin incision.

The medial femoral condyle is continually exposed to physiological shearing stress during knee motion positions. So, biplanar stresses can lead to a fixation failure in the condyle [4]. They vary considerably in view of the surgical approach in treating coronal plane fractures of the femoral condyle [6]. It is generally accepted that screw fixation is a good fixation method for treating Hoffa fractures [7,9]. Tetsunaga et al. applied a technique of placing a posterior buttress plate with a lateral compression plate to enhance the stability of the Hoffa fracture [16]. Nandy et al. described a sandwich technique using an interposition bone graft, a lag screw, and a neutralization plate for a non-union medial Hoffa fracture, and they obtained a good functional outcome [6]. Jarit et al., in a biomechanical study, showed that the placement of screws in a posteroanterior direction gives more stability than anteroposteriorly placed lag screws [17]. However, posterior plate and screw placement are technically difficult, and there is also a risk of osteonecrosis due to the disruption of the intraosseous blood supply to the medial femoral condyle.

Borse et al. demonstrated the use of headless compression screws in these fractures [18]. Generally, 3.5-mm or 4.5-mm screws were recommended for these fractures [4]. We used 4.5-mm headless compression screws to make a different configuration from the anterior to the posterior direction of the medial femoral condyle and from the non-articular medial surface to the lateral direction. This way, the crossed screws are more rigid, especially in resisting torsional stresses, and they provide better stabilization [9]. Hak et al. showed that there is a need to use at least 2×3.5-mm screws to approximate the biomechanical stability of a single 6.5-mm screw for the fixation of coronal plane fractures of the femoral condyle [19].

Xu et al. described a fixation of a type III coronal plane fracture of the femoral condyle using three screws. One screw is inserted from the femoral intercondylar notch, and the other two screws are inserted from the non-articular lateral or medial surface of the fractured condylar fragment [9]. They reported that this method may be more suitable for Type III Hoffa fractures [9]. Iwai et al. reported a case of using intra-articular corrective osteotomy and the elevation of the articular cartilage for a malunited coronal plane fracture of the lateral femoral condyle with a good outcome [8].

Sasidharan et al. presented a case of malunion of a medial condyle fracture in the coronal plane, which developed after an operation of the Hoffa fracture in a patient [4]. In our study, the malunion of the medial condyle fracture in the coronal plane developed after the neglected fracture. Sasidharan et al. used two cannulated cancellous screws introduced in the anteroposterior direction for osteogenesis [4]. They reported that intra-articular osteotomy was a feasible salvage option for a malunited medial Hoffa fracture with a moderate improvement in the range of motion of the knee and a reasonable outcome [4].

Some authors recommended an immediate, unrestricted range of motion of the knee following surgery [6,9,15]. Lewis et al. suggested immobilization in full extension for six weeks in case of concern regarding the stability of fixation [14]. Sasidharan et al. suggested that weight-bearing walking should be delayed for two months to allow fracture consolidation [4]. The fact that lag screws alone cannot counteract the biplanar deforming forces on the construct should be considered [4,16,19]. Therefore, we made immobilization in a cylinder cast for two weeks compulsory postoperatively, and patients were allowed to bear weight as tolerated after two months.

## Conclusions

Corrective osteotomy for a malunited medial Hoffa fracture is technically even more challenging. Therefore, proper diagnosis and accurate operative treatment are very important to prevent the malunion of Hoffa fractures. However, if malunion at the fracture site occurs, intra-articular corrective osteotomy should be considered as a salvage treatment to prevent poor outcomes.
